# Genomics in Public Health: Perspective from the Office of Public Health Genomics at the Centers for Disease Control and Prevention (CDC)

**DOI:** 10.3390/healthcare3030830

**Published:** 2015-09-15

**Authors:** Ridgely Fisk Green, W. David Dotson, Scott Bowen, Katherine Kolor, Muin J. Khoury

**Affiliations:** 1 Carter Consulting, Inc. and Office of Public Health Genomics, Centers for Disease Control and Prevention, Atlanta, GA 30333, USA; 2 Office of Public Health Genomics, Centers for Disease Control and Prevention, Atlanta, GA 30333, USA; E-Mails: gle1@cdc.gov (W.D.D.); Msb4@cdc.gov (S.B.); Bqx7@cdc.gov (K.K.); Muk1@cdc.gov (M.J.K.)

**Keywords:** public health genomics, public health, genomics, genetics, epidemiology, precision medicine, horizon scanning, guidelines, Office of Public Health Genomics, Centers for Disease Control and Prevention (CDC)

## Abstract

The national effort to use genomic knowledge to save lives is gaining momentum, as illustrated by the inclusion of genomics in key public health initiatives, including *Healthy* *People* *2020*, and the recent launch of the precision medicine initiative. The Office of Public Health Genomics (OPHG) at the Centers for Disease Control and Prevention (CDC) partners with state public health departments and others to advance the translation of genome-based discoveries into disease prevention and population health. To do this, OPHG has adopted an “identify, inform, and integrate” model: identify evidence-based genomic applications ready for implementation, inform stakeholders about these applications, and integrate these applications into public health at the local, state, and national level. This paper addresses current and future work at OPHG for integrating genomics into public health programs.

## 1. Introduction

The increasing prioritization of genomics in public health is reflected in the inclusion of genomics in key public health initiatives. Pathogen genomics has been used in public health for decades and has been integrated into Centers for Disease Control and Prevention (CDC) activities, most recently through the Advanced Molecular Detection Initiative, which addresses the applications of pathogen genomics methods and technologies [1]. For this article, the focus will be on human genomics. *Healthy* *People* *2020* includes Genomics as a new topic area, with the following objectives: (1) increase the proportion of women with a family history of breast and/or ovarian cancer who receive genetic counseling; and (2) increase the proportion of persons with newly diagnosed colorectal cancer who receive genetic testing to identify Lynch syndrome (or familial colorectal cancer syndromes) [2]. *BRCA* genetic counseling is a preventive service covered by many insurers under the Affordable Care Act in accordance with the U.S. Preventive Services Task Force (USPSTF) recommendation [3,4,5]. Stage 2 of the Medicare and Medicaid Electronic Health Record Incentive Programs include the requirement that more than 20% of patients have structured data entries for family health history for one or more first-degree relatives [6]. The Precision Medicine Initiative aims to provide tailored clinical care based on individuals’ unique characteristics, including their genome, microbiome, family health history, and behaviors [7]. “Precision public health” approaches can bring a population-based perspective that complements the individualized clinical focus [8]. Furthermore, precision medicine relies on public health by utilizing large repositories of health data, such as public health disease registries, to interpret individual results.

Since its inception in 1997, the Office of Public Health Genomics (OPHG) at the CDC has worked with state health departments and other partners to identify opportunities for genomics to improve population health and provided foundational contributions to help support genomics-related public health activities. These activities include establishment of the Evaluation of Genomic Applications in Practice and Prevention (EGAPP) Working Group, whose recommendations on Lynch syndrome are cited in *Healthy* *People* *2020* [9], and work building the evidence base for family health history in public health [10,11]. Current activities at OPHG support the *Healthy* *People* *2020* goals of increased identification and provision of genetic services for individuals at risk for Hereditary Breast and Ovarian Cancer (HBOC) and Lynch syndrome and help define the role of “precision public health.” OPHG works to promote effective and responsible translation of genome-based discoveries into disease prevention and population health. To do this strategically, OPHG has adopted an “identify, inform, and integrate” model: identify evidence-based genomic applications ready for implementation, inform stakeholders about these applications, and integrate these applications into public health at the local, state, and national level. In this paper, we will describe current and future work at OPHG and how these projects contribute to the “identify, inform, and integrate” model. This work includes activities based on horizon scanning, guidelines, informatics, and support of state genomics programs.

## 2. Identifying and Assessing Opportunities for Genomics to Impact Public Health

OPHG uses a systematic literature research method called horizon scanning [12,13], which aids in identifying genomics-related research on evidence-based translation of genomic discoveries into improved health care and disease prevention that have a potential impact on population health. Horizon scanning identifies both scientific literature and popular press items. OPHG creates and disseminates a Weekly Update that includes articles identified through the horizon scanning process. One key focus of the process is to identify guidelines relating to genomics and health. Evidence-based guidelines help inform the implementation of genomic technologies in clinical and public health practice, and will be critical to establish the credibility of the recently launched precision medicine initiative. Guidelines identified through OPHG horizon scanning are cataloged online in the Tier Classification of Genomic Applications by Levels of Evidence [14,15,16]. Tier 1 genomic and family health history applications have a base of synthesized evidence supporting implementation into practice. Tier 2 genomic and family health history applications have synthesized evidence that is insufficient to support routine implementation in practice; however, existing evidence may provide information for informed decision making by providers and patients. Tier 3 genomic and family health history applications either have synthesized evidence culminating in recommendations against use (or discouraging use), OR no relevant synthesized evidence was identified. Tier 3 applications are not ready for routine practice, but may be considered in clinical and population research. Work to incorporate these applications into a publically available, interactive database is currently in progress. The database will also include scientific literature identified through horizon scanning.

Promoting the need for a robust evidence base for genomic applications has been a consistent theme for OPHG. Inspiration was drawn from the successes of groups such as the US Preventive Services Task Force, The Guide to Community Preventive Services, The Cochrane Collaboration, and many others, as powerful indicators for the value of evidence-based evaluation to support rational integration of genomics in health. The ACCE (Analytic validity, Clinical validity, Clinical utility, Ethical, legal, and social implications) model process was an early initiative supported by OPHG to promote the development and use of targeted and balanced methods in evidence synthesis [17]. Although not the first source to coin the term “clinical utility”, ACCE was seminal in emphasizing how the term could best be defined in the context of genetic test evaluation, and where it fits in relation with other common components of evaluation. ACCE defined clinical utility of genetic testing in terms of improved patient outcomes, while encompassing the validity and societal aspects of ACCE as well.

OPHG sponsored the EGAPP initiative to put the lessons learned from development of the ACCE model into action. In addition to the ACCE criteria, EGAPP also considers clinical factors, test availability, cost effectiveness, and other contextual factors in making recommendations. EGAPP is an ongoing effort to develop and apply evidence-based evaluation practices in the field of genomic health. The initiative has been centered on the independent EGAPP working group, which has developed, refined, and tested a plethora of methods for evaluation of genomic tests. Since 2007, the EGAPP working group has published nine recommendation statements, three methods reports, and an article describing the lessons they have learned throughout the process [18]. EGAPP recommendations, such as the 2009 recommendation for universal Lynch syndrome screening for people with newly diagnosed colorectal cancer, have informed national guidelines, institutional screening protocols, and health insurance coverage determinations [19,20,21].

## 3. Toward Realizing Population Health Benefits

Extending the foundational work of EGAPP toward realizing population health benefits of evidence-based recommendations requires new efforts to bring public health, health care, and other stakeholders together to: (1) accelerate research translation and implementation of genomics; (2) develop and apply methods for assessing best practices and outcomes for implementation of genomic applications and their impact in the real world; (3) assemble and enhance metrics, tools, and education materials that can increase uptake of genomic applications in health care and disease prevention and for informing and engaging providers, policy makers, and the general public; and (4) develop pilot projects for implementation of genomic applications at the interface of public health and healthcare, with a focus on surveillance and measurement, policy impact, education, and programs.

State public health departments are essential partners in the national effort to use genomic knowledge to save lives. CDC has provided both financial and technical support to states implementing genomics approaches to public health. In 2003, the year of the completion of the Human Genome Project, CDC issued five-year cooperative agreements with the purpose of integrating genomics into state based public health policy and programs. The four awardees, Michigan, Minnesota, Oregon, and Utah, pursued this goal successfully using different approaches. These states soon became pioneering examples for generalizable public health genomics implementation efforts which included: (1) promoting planning both internally and with outside partners to use genomic information, including genetic testing and family history data, in existing policy and programs; (2) developing workforce capacity and leadership for application of genomic knowledge and technologies to chronic, infectious, and environmental diseases; (3) establishing leadership for genomics integration into plans and prevention programs for asthma, cancer, cardiovascular disease, and diabetes; and (4) developing evidence for the use of genomics in disease prevention and established sustainable interventions involving family health history collection, assessments, and educational curricula and materials for the health workforce, policymakers, and the public [22].

When this trailblazing agreement was completed, OPHG, CDC issued new three-year cooperative agreements in 2008 which built upon the infrastructure developed in the previous cycle, while focusing on opportunities to use public health strengths of surveillance, education, and policy development to promote and monitor implementation of newly emerging evidence-based genomic testing and family health history recommendations. Activities focused on supporting implementation of the 2005 USPSTF *BRCA* recommendation, with states developing model programs such as using cancer registry data to educate health care providers and institutions about patients in their care who could benefit from evidence-based genetic testing recommendations [22]. Michigan and Oregon were the awardees.

Model state activities similar to those initiated under the 2008 CDC cooperative agreements received support through two Division of Cancer Prevention and Control (DCPC), CDC funding opportunities awarded in 2011 and 2014 to Michigan, Oregon, Georgia, and Utah [23]. In addition, Connecticut received funding support through a 2011 *Healthy* *People* *2020* Action Award.

These CDC-funded state activities served as model programs and informed the development of resources and tools to assist other states in implementing genomics approaches in public health. These tools include the State Implementation Activities Clickable Map and the Genomics Toolkit. The State Implementation Activities Clickable Map provides state-by-state information on activities related to implementation of three Tier I public health genomics applications: Hereditary Breast and Ovarian Cancer (HBOC), Lynch Syndrome, and Familial Hypercholesterolemia (FH), as well as general information on state public health genomics programs. The Tier 1 Genomic Applications Toolkit for Public Health Departments describes genomics approaches used by model state programs. The Toolkit outlines key first steps that state public health departments can take to identify existing state priorities and activities related to genomics. These steps include identifying objectives related to Tier 1 applications in the state comprehensive cancer control plan and heart disease and stroke plan, reviewing existing Tier 1 activities and resources in the state using the State Implementation Activities Clickable Map, and choosing projects based on the successful examples provided in the Toolkit. Approaches discussed in the Toolkit are divided into Phase 1 and Phase 2. Phase 1 approaches focus on identification of individuals who could benefit from Tier 1 genomic applications, while Phase 2 approaches expand to finding at-risk family members of these individuals through cascade screening. These risk identification activities fulfill a major aim of precision public health. The Toolkit includes basic information on each of the three Tier 1 conditions and the current Tier 1 recommendation. The Toolkit provides examples of Phase 1 approaches implemented by state health departments: (1) use of cancer registry data to identify individuals at high risk for Lynch syndrome and Hereditary Breast and Ovarian Cancer syndrome (one form of bi-directional cancer registry reporting); (2) informing policy making, such as evidence-based coverage by payers for services specified by Tier 1 recommendations; (3) developing and tracking surveillance indicators to follow progress in achieving widespread implementation of Tier 1 recommendations and the *Healthy* *People* *2020* objective; and (4) designing and implementing educational outreach programs targeting the general public and health professionals about Tier 1 applications. A limited number of Phase 2 approaches are described. Tools and educational materials developed by OPHG and states are listed. The section on Lynch syndrome includes state-by-state estimates of the number of people with Lynch syndrome who could be identified by screening all persons with newly diagnosed invasive colorectal cancer.

OPHG recently partnered with Genetic Alliance [24] to develop materials to support states in implementing bi-directional cancer registry reporting to identify individuals at high risk for Lynch syndrome and HBOC. Bi-directional cancer registry reporting is a mechanism by which two-way communication occurs between state cancer registries and the institutions that report patient information to them. To identify individuals at high risk for Lynch syndrome and HBOC, the state cancer registries use the routine data submitted to them by providers and institutions for cancer surveillance purposes and apply national guidelines to that information. Such guidelines include the EGAPP guidelines for Lynch syndrome and USPSTF guidelines for HBOC. The state cancer registries then report back to institutions and providers that risk, along with information that helps providers and patients make decisions about next steps for clinical care that can improve health outcomes and reduce the risk of patients developing cancers. The materials developed to support bi-directional reporting include a video geared toward providers and institutions describing bi-directional cancer registry reporting to identify individuals at high risk for Lynch syndrome and HBOC and a slide set describing HBOC and Lynch syndrome screening, diagnosis, and management in detail. Written materials developed include fact sheets on HBOC and Lynch syndrome for providers and patients, sample bi-directional reporting data forms, summaries of current recommendations on screening for HBOC and Lynch syndrome, an FAQ on cancer registries, and a sample form that states can use to list local genetic specialists. In addition, materials were developed to support individuals diagnosed with HBOC or Lynch syndrome in informing their family members about their condition. These materials consist of sample letters to send to family members that describe the syndrome, the implications for family members, and suggested next steps, and a pamphlet with suggestions on how patients can talk to family members about their diagnosis. These materials were based on those developed by Michigan and Connecticut for their bi-directional reporting programs and in response to feedback received from states that had already implemented or were planning to implement bi-directional cancer registry reporting to identify individuals at high risk for Lynch syndrome and HBOC programs.

## 4. Conclusions

With continued advances in genomics, we are seeing an increasing number of genomic applications that can be used in public health programs. Through the ongoing activities summarized above, OPHG is able to fulfill its mission to identify, inform, and integrate evidence-based genomic testing and family health history applications to realize health impact. The horizon scanning process identifies genomic applications at different stages of translation. The database currently in development will provide a valuable resource to inform stakeholders about key applications in genomics, with searchable databases for guidelines, articles on translation of genomic applications, human genome epidemiology literature, CDC resources, and other items. The Tier Classification of Genomic Applications by Levels of Evidence and Weekly Update serve to inform key stakeholders about genomic approaches and their readiness for implementation. OPHG supports integration of those applications ready for translation through EGAPP and similar initiatives and by providing resources to states such as the clickable map and toolkit.

OPHG is actively exploring emerging opportunities to integrate genomic applications in public health. OPHG has written a journal article [25] and blogs [8,26,27,28] defining the role for public health in the precision medicine initiative. “Precision Public health” approaches include the use of genomics in the investigation and control of infectious diseases, the use of information technologies and data science in enhancing the precision and speed of public health surveillance and tracking, and use of genomics and family health history to target disease prevention to subsets of the population at high risk [8]. OPHG is exploring the potential public health role for promoting cascade screening. OPHG encourages the use of family health history for risk assessment for chronic conditions, while also considering current and future uses for genomics in chronic disease risk assessment as well as in treatment through pharmacogenomics and other applications. Through these activities, OPHG will continue to support the optimal integration of genomic advances into public health.

## References

[1] Advanced Molecular Detection (AMD). http://www.cdc.gov/amd/index.html.

[2] Healthy People 2020: Genomics. http://www.healthypeople.gov/2020/topics-objectives/topic/genomics.

[3] Recommendation Summary. U.S. Preventive Services Task Force. http://www.uspreventiveservicestaskforce.org/Page/Topic/recommendation-summary/brca-related-cancer-risk-assessment-genetic-counseling-and-genetic-testing.

[4] Preventive Services Covered Under the Affordable Care Act. http://www.hhs.gov/healthcare/facts/factsheets/2010/07/preventive-services-list.html.

[5] FAQS about Affordable Care Act Implementation—Coverage of BRCA Testing. http://www.dol.gov/ebsa/faqs/faq-aca26.html.

[6] Centers for Medicare and Medicaid Services: Stage 2. http://www.cms.gov/Regulations-and-Guidance/Legislation/EHRIncentivePrograms/Stage_2.html.

[7] The Precision Medicine Initiative. https://www.whitehouse.gov/precision-medicine.

[8] Khoury M.J. CDC Public Health Genomics: Precision Public Health and Precision Medicine: Two Peas in a Pod. http://blogs.cdc.gov/genomics/2015/03/02/precision-public/.

[9] Evaluation of Genomic Applications in Practice and Prevention Working Group (2009). Recommendations from the EGAPP working group: Genetic testing strategies in newly diagnosed individuals with colorectal cancer aimed at reducing morbidity and mortality from Lynch syndrome in relatives. Genet. Med..

[10] Yoon P.W., Scheuner M.T., Peterson-Oehlke K.L., Gwinn M.L., Faucett A., Khoury M.J. (2002). Can family history be used as a tool for public health and preventive medicine?. Genet. Med..

[11] Yoon P.W., Scheuner M.T., Jorgensen C., Khoury M.J. (2009). Developing Family Healthware, a family history screening tool to prevent common chronic diseases. Prev. Chronic Dis..

[12] Clyne M., Schully S.D., Dotson W.D., Douglas M.P., Gwinn M., Kolor K., Wulf A., Bowen M.S., Khoury M.J. (2014). Horizon scanning for translational genomic research beyond bench to bedside. Genet. Med..

[13] Gwinn M., Grossniklaus D.A., Yu W., Melillo S., Wulf A., Flome J., Dotson W.D., Khoury M.J. (2011). Horizon scanning for new genomic tests. Genet. Med..

[14] CDC Public Health Genomics: Guidelines, Policies and Recommendations in Genomics. http://www.cdc.gov/genomics/gtesting/guidelines.htm.

[15] CDC Public Health Genomics: Genomic Tests and Family Health History by Levels of Evidence. http://www.cdc.gov/genomics/gtesting/tier.htm.

[16] Dotson W.D., Douglas M.P., Kolor K., Stewart A.C., Bowen M.S., Gwinn M., Wulf A., Anders H.M., Chang C.Q., Clyne M. (2014). Prioritizing genomic applications for action by level of evidence: A horizon-scanning method. Clin. Pharmacol. Ther..

[17] CDC Public Health Genomics: ACCE Model Process for Evaluating Genetic Tests. http://www.cdc.gov/genomics/gtesting/ACCE/.

[18] Evaluation of Genomic Applications in Practice and Prevention (EGAPP). http://egappreviews.org.

[19] Blue Cross Blue Shield of North Carolina Corporate Medical Policy: Genetic Testing for Colon Cancer. http://www.bcbsnc.com/assets/services/public/pdfs/medicalpolicy/genetic_testing_for_colon_cancer.pdf.

[20] National Comprehensive Cancer Network NCCN Clinical Practice Guidelines in Oncology. Genetic/Familial High-Risk Assessment: Colorectal. Version 1.2015. Fort Washington (PA). http://www.nccn.org/professionals/physician_gls/pdf/genetics_colon.pdf.

[21] Giardiello F.M., Allen J.I., Axilbund J.E., Boland C.R., Burke C.A., Burt R.W., Church J.M., Dominitz J.A., Johnson D.A., Kaltenbach T. (2014). Guidelines on genetic evaluation and management of lynch syndrome: A consensus statement by the U.S. multi-society task force on colorectal cancer. Am. J. Gastroenterol..

[22] St Pierre J., Bach J., Duquette D., Oehlke K., Nystrom R., Silvey K., Zlot A., Giles R., Johnson J., Anders H.M. (2014). Strategies, actions, and outcomes of pilot state programs in public health genomics, 2003–2008. Prev. Chronic Dis..

[23] Trivers K.F., Rodriguez J.L., Cox S.L., Crane B.E., Duquette D. (2015). The activities and impact of state programs in public health genomics, 2011–2014. Healthcare.

[24] Genetic Alliance. www.geneticalliance.org.

[25] Khoury M.J., Evans J.P. (2015). A public health perspective on a national precision medicine cohort: Balancing long-term knowledge generation with early health benefit. JAMA.

[26] Khoury M.J. CDC Public Health Genomics: Precision Medicine and Public Health: Improving Health Now While Generating New Knowledge for the Future. http://blogs.cdc.gov/genomics/2015/06/02/precision/.

[27] Khoury M.J., Richardson L.C. CDC Public Health Genomics: Using Genomics in Precision Prevention of Breast Cancer. http://blogs.cdc.gov/genomics/2015/04/23/using-genomics/.

[28] Khoury M.J. CDC Public Health Genomics: When a Country Cannot be a Cohort: Challenges of Implementing a Large Precision Medicine Cohort Study in the United States. http://blogs.cdc.gov/genomics/2015/03/23/when-a-country/.

